# Comprehensive clinical and pathological analysis of three rare vascular tumor cases

**DOI:** 10.3892/ol.2012.1025

**Published:** 2012-11-13

**Authors:** FU LI, LIXIA MA, LELING ZHANG, LIBO ZHENG, XIN LV, JIHUA FU

**Affiliations:** 1 Pediatric Research Institute, Qilu Children’s Hospital, Shandong University, Jinan 250022, P.R. China; 2 Department of Pediatric Hematology, Qilu Children’s Hospital, Shandong University, Jinan 250022, P.R. China

**Keywords:** vascular tumors, clinical, pathological

## Abstract

The histological boundary between benign and malignant vascular tumors is not clear. Thus, the discrepancies between clinical judgement and pathological diagnosis often lead to a difficult clinical decision, and may result in misdiagnosis. In order to develop more effective treatment methods, the clinical and pathological data concerning rare vascular tumors should be comprehensively analyzed. To clarify the important roles of clinical and pathological analyses in vascular tumors, three rare vascular tumor cases that we encountered in clinical practice are analyzed and reported in detail.

## Introduction

Vascular tumors, which derive from vascular tissue, mostly occur in the head, neck and subcutaneous tissue of the limbs and muscles. Generally, different types of vascular tumors occur in different age brackets. For example, skin tumors always occur in young adults, and the peak age of these patients is 20–30 years of age; while endothelial tumors often appear in older individuals, mainly in their 40s. Moreover, there are no obvious differences in this phenomenon between males and females. Large lumps, accompanied by local pain, dysfunction or other symptoms, are the obvious pathological features of patients with vascular tumors. Although it is easy to diagnose the rare vascular tumors by pathological analysis due to the visible histological features, the boundary definition between benign and malignant tumors in histology is still a problem that needs to be addressed. The discrepancies between clinical judgement and pathological diagnosis often lead to a difficult clinical decision, which may result in misdiagnosis. In this context, comprehensive analyses of clinical and pathological data of rare vascular tumors are necessary.

Vascular tumor specimens were fixed in 10% neutral buffered formalin, embedded by paraffin, cut into 4-*μ*m-thick serial sections, and then stained with HE and reticular fiber. For the immunohistochemical staining, the Envision™ two-step staining protocol was adopted. Briefly, after antigen retrieval in pH 6.0 10 mmol/l citrate buffer for 20 mins, the sections were prepared by conventional dewaxing hydration procedures. In this report, the analysis data of three rare vascular tumor cases that we encountered in clinical practice are reported. The study was approved by Ethics Committee of Qilu Children’s Hospital of Shandong University, Jinan, China. Written informed consent was obtained from the patient’s family.

## Case report

### Case 1: Angiosarcoma

**Figure 1.**
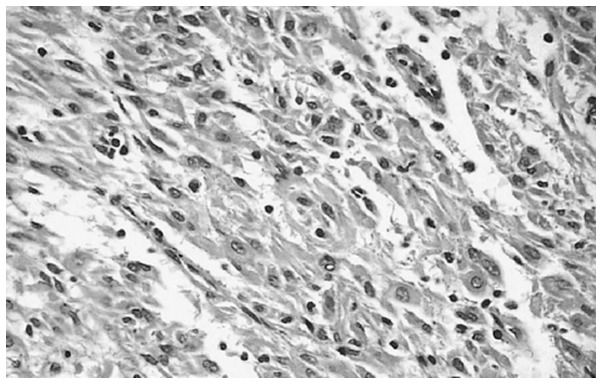
The irregular tumor blood vessels in a hepatic angiosarcoma patient.

**Figure 2.**
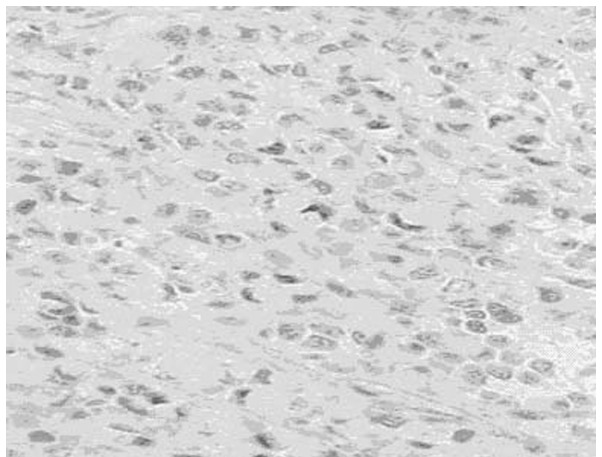
The division of the vessel lumen in single cells in a hepatic angiosarcoma patient.

**Figure 3.**
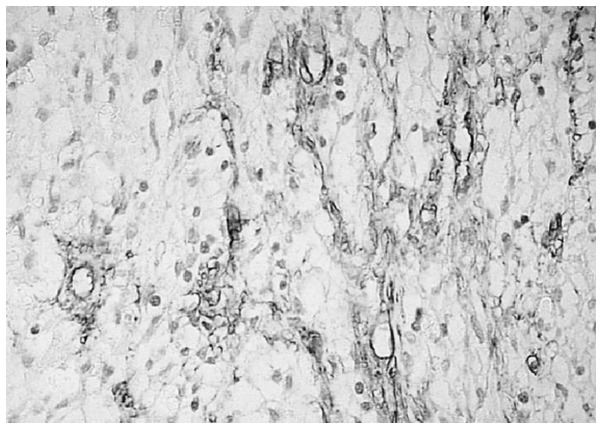
The expression of nestin in a hepatic angiosarcoma patient.

The patient was a 47-year-old male who was reported to have a potential medical B-liver cancer, according to the clinical symptoms and laboratory tests. However, further examination was still required to confirm the diagnosis prior to surgery, since the symptoms and tests were not specific enough. The postoperative pathology revealed a hepatic angiosarcoma (HAS). Pathological examination showed the vascular tumors located in the right lobe of the liver with solid sections, hemorrhage, necrosis and medium texture. The boundary between tumors and surrounding liver tissue was unclear, and there were incomplete fibrous capsules around tumors. The liver biopsy specimen exhibited light brown spinal cord-like tissue. Microscopic examination showed that the tumor tissue consisted of irregular tumor blood vessels that formed mesh compartments, and were lined with one or more layers of endothelial cells. Endothelial cell proliferations projecting out of the cavity were papillary (Fig. 1). Hemorrhage and necrosis were observed in the tumors. Surgically-resected tumor tissue centers on the hardening zone were rich in fiber and accompanied with hyaline degeneration. There were many tumor cells in the marginal zone with peripheral enhancement, which was consistent with the findings from the CT and MRI scans, and scattered with visible residue and proliferation of small bile ducts around them. Certain tumor cells had an irregular shape, such as circular, polygonal or epithelioid, with rich cytoplasm. These cells demonstrated atypical cell division that was difficult to see. RBCs, which divide the vessel lumen into single cells, were observed in the cytoplasm of some tumor cells under closer examination (Fig. 2). Immunohistochemistry revealed the expression of nestin (Fig. 3).

### Case 2: Kaposiform hemangioendothelioma (KHE)

**Figure 4.**
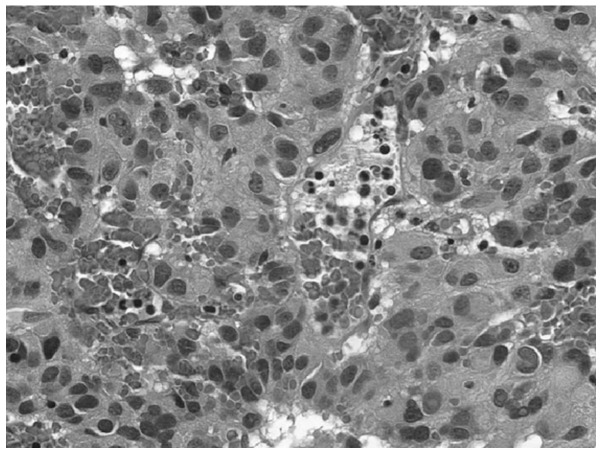
Fusiform endothelial cells in a Kaposiform hemangioendothelioma patient.

**Figure 5.**
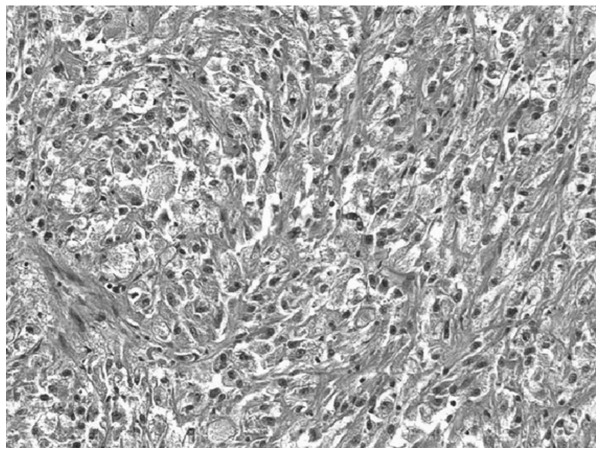
The expression of CD34 and CD31 in a Kaposiform hemangioendothelioma patient.

The patient was a 5-month-old child, who was hospitalized due to swelling of the right subaxillary for 2 days. Physical examination showed a 3x4 cm right, subaxillary lump with a light red surface. B-mode ultrasonic imaging showed an 8×6×2 cm solid, slightly enhanced echo mass of the armpit with a clear border. The blood routine results were: WBC, 12.8×10^9^/l; RBC, 2.15×10^12^/l; PLT, 42×10^9^/l; and fibrinogen was decreased by 23 mg/ml. The mass in the deep subcutaneous tissue was ill-defined and locally associated with distended lymphatic vessels containing yellow lymph. Pathological examination showed a section of skin tissue (4.3×3.9×2.1 cm), which was pale brown, nodular and of medium texture. It partially consisted of small pieces of sponge-like lumen containing yellow fluid. Microscopic examination showed that the tumor tissue consisted of fusiform endothelial cells and some round nuclei of epithelioid form (Fig. 4). The tumors were nodular in appearance, and contained a small amount of glomerular-like structures. Endothelial cells of local, crack-like lumen proliferating in a papillary form and a small transparent thrombosis were observed. At the edge, there was a crack-like lymphangioma structure; tumor tissue showed multiple nodular forms infiltrating to intramuscular. Immunohistochemistry revealed that spindle tumor cells and vascular endothelial cells all expressed CD34 and CD31 (Fig. 5).

### Case 3: Pleomorphic hyalinizing angiectatic tumor

**Figure 6.**
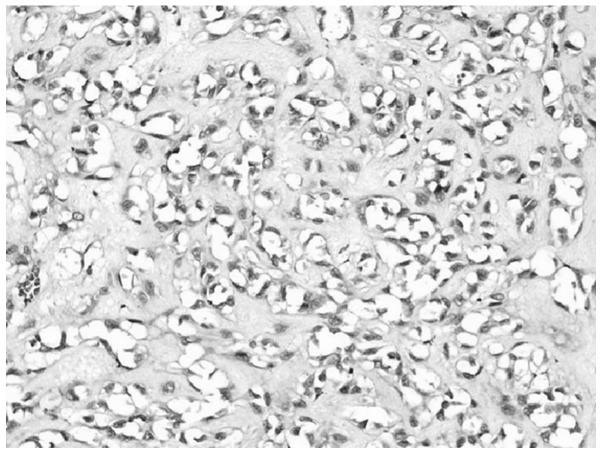
The collagen fibers.

**Figure 7.**
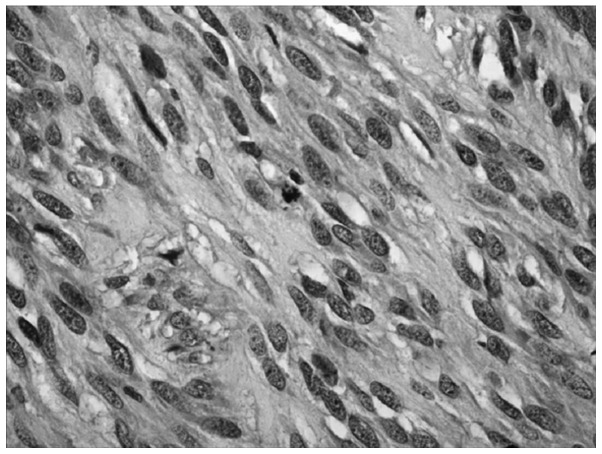
The expressed CD34.

The patient was a 30-year-old woman, who was hospitalized due to a right inguinal lump which had persisted for more than six months. Physical examination revealed an egg-sized mass in the right inguinal region, with a clear border, good activity and no tenderness. The preliminary clinical diagnosis was skin fibroma. Surgical resection showed the mass was located subcutaneously with no adhesion to the surrounding tissue. Pathological examination showed that the tumor was an oval, 6×5×5 cm large, thin and complete capsule, with a solid section, white color and a slightly hard texture. A small part of the cavity contained fissures of different sizes, which contained a small amount of red liquid. Microscopic examination showed that the tumor was mainly composed of thin-walled blood vessels and fibrous tissue lumen of different sizes, with hyalinosis of the wall and around collagen fibers (Fig. 6). Spindle endothelial cells were lined with expanding blood vessels. There were a small amount of RBCs in the cavity and a thrombus in part of the lumen. In between the vessels, there were tumor cells of multiple forms, including hyperchromatic nuclei and clear nucleolus. Some of the tumor cells showed marked atypia and giant tumor cells showing no mitotic activity were observed. Immunohistochemistry results were as follows: CD34 (+), S-100 (-), CK (-), SMA (-) (Fig. 7). The pathological diagnosis was soft tissue pleomorphic hyaline expansion of tumor blood vessels.

## Discussion

Hepatic angiosarcoma (HAS) is an uncommon neoplasm of endothelial cells of hepatic sinusoids. However, it is the most common malignant mesenchymal tumor of the liver. It is more common in males in late adulthood. The etiology of most cases of primary HAS is unknown; some have been associated with exposure to vinyl chloride, thorium dioxide, arsenic, anabolic steroids, and diseases such as hemachromatosis and von Recklinghausen neurofibromatosis (1). The diagnosis of HAS is often performed too late due to lack of specific symptoms, laboratory tests and radiological findings. Therefore, it is necessary to make more detailed observations of the morphology and immunohistohemical staining on the basis of the clinical manifestations.

To investigate the value of assisted diagnosis of the malignant vascular tumors, we conducted a nestin test on patient 1. Studies show the expression of nestin in vascular endothelial cells (2,3). The designation ‘nestin’ refers to a member of the family of intermediate filaments and comes from the fact that this protein is expressed mainly in neuroepithelial stem cells and is of vascular origin (4–6). The result showed positive expression of nestin in the cytoplasm and we found that the expression of nestin in malignant vascular tumors was related to the shape change of the tumor. When the tumor cells were spindle-shaped, strong expression as well as deep stained cytoplasm was found. When the tumor cells were epithelioid, there was weak or even no expression of nestin. However, nestin is not expressed in mature elements and terminal cell differentiation is associated with loss of immunoreactivity to this protein. It is possible that spindle cells are in a dedifferentiated, immature state and are therefore nestin-positive. Thus, the presence of nestin may indicate dedifferentiation in these tumors as well (7). Therefore, we should continue to observe and analyze the assisted diagnostic value of nestin in malignant vascular tumors in the future. In addition, HAS is often confused with other spindle tumor cells. However, these tumor cells were arranged in bundles or interwoven shapes and immunohistohemical endothelial cell markers were negative.

Kaposiform hemangioendothelioma (KHE) is a rare and aggressive vascular tumor, which mainly occurs in infants and children as first proposed in a study by Zukerberg *et al*(8). It usually presents clinically as angioma with thrombocytopenia and low fiber albumin, namely thrombocytopenic purpura syndrome (Kasabach-Merritt syndrome, KMS). This feature differentiates this entity from a juvenile hemangioma that forms the closest differential diagnosis. It is usually identified in infancy and in the first decade of life at sites such as extremities and the retroperitoneum, and is uncommonly found in the head and neck region (9–11). At times, KHE occurs without KMS (12). It has rarely been documented in the subaxillary.

Case 2 was identified to be clinically manifested as KMS and the pathological diagnosis was KHE. The child was treated with partial resection, corticosteroid and pingyangmycin local injection for residual tumor, VCR and CTX chemotherapy. As a result, the tumor shrank and continued to do so for six months. The child survived with the tumor and PLT rose to 100×10^9^/l. Enjolras *et al*(13) believed that the infant hemangioma associated with KMS was not ordinary hemangioma; both histological results showed KHE or tufted hemangioma. Sarkar *et al*(14) reported 21 cases of KMS and all cases were diagnosed as KHE, indicating that KHE was the main pathological form of KMS hemangioma. On the other hand, the pathological diagnosis of KHE, whether it be thrombocytopenia or low fibrinogen disease, should be considered in the clinical analysis. Overall incidence of KHE is not high and there is no consensus on its treatment. Currently, complete resection of the tumor is considered as the best treatment method and the prognosis is improved by increased resection of superficial tumors. Also, after complete resection, no tumor survived within 2 or 3 years. In addition, the disease is easily confused with Kaposi’s sarcoma; both diseases contain spindle cells and slit like blood vessels, while the latter was infiltrated by inflammatory cells without glomerular-like structures and commonly occurred in adults.

Pleomorphic hyalinizing angiectatic tumor (PHAT), a rare neoplasm of unknown origin, an independent entity of soft tissues, is a type of non-metastasizing tumor (15). Some immunohistochemical and ultrastructural studies indicate that the neoplastic cells may be a type of undifferentiated, primitive mesenchymal cell, possibly related to stromal fibroblasts (16). This disease was firstly described by Smith *et al* disease in 1996 (17) and 14 cases were reported. The disease was mainly found in the trunk, limbs and subcutaneous tissue of lower limbs in adult women. It generally appeared as a mass of slow growth (18). The histological features were thin-walled and expanded blood vessels that were distributed in clusters; the walls and matrix transparently degenerated and residual pleomorphic tumor was visible among blood vessels with the split as low as <1/50 HPF. Immunohistochemistry showed expression of CD34 and S-100. This was a low-grade malignant tumor. Out of 14 cases, only 4 cases had recurrence, and no metastasis was found. The case had the same features in clinical, histological and immunohistohemical analysis as mentioned above, which was consistent with this diagnosis. The most difficult, differential diagnoses of PHAT are neurilemoma and malignant fibrous histiocytoma (19,20). Neurilemomas may also occur with angiectatic and hyalinized vasculature, cells with prominent pseudonuclear inclusions and pleomorphic cells as observed in ancient or degenerated neurilemomas; however, they are encapsulated, have characteristic Antoni A and B zones and are nearly all strongly reactive to S-100 protein. Low mitotic rates, clusters of hyalinized thin-walled ectatic vessels, and immunoreactivity for CD34 help differentiate PHAT from malignant fibrous histiocytoma. However, since the study was limited, the biological behavior remains to be studied. Meanwhile, due to the complexity and diversity of forms of the disease’s pathology, a variety of benign and malignant tumors need to be identified. However, these need to be identified according to the result that the disease has clear cellulose thin-walled blood vessels and vascular calm between the more spindle-shaped interstitial cells of the typical characteristics of immunohistochemical expression of Vim and VEGF, but not the expression of SMA, Des, CK and CD31, for example. Subsequently, we could exclude the other tumors.

Vascular tumors, although occurring relatively rarely in clinical practice, are a multi-disciplinary disease, which is easily ignored by clinicians. The clinical manifestations and the gross specimens of this tumor are often hard, tough and resemble fish flesh. Mainly by pathological diagnosis of the vascular tumor, body surface or body cavity for the rapid growth of tumor recurrence if the transfer should be taken into account the possibility of such tumors. Signs of suspected malignancy could puncture cytology during surgery or before the rapid intraoperative pathological examination. Once diagnosed, resection should be appropriately extended. For vascular tumors, surgery is the primary treatment method and radiation therapy has a significant effect in preventing recurrence. If a comprehensive clinical and pathological analysis is carried out and an accurate diagnosis is made, early detection and treatment may reduce the pain of the patients. Therefore, for a rare vascular tumor, the clinical and pathological data must be comprehensively analyzed.
